# Supplementary material for: The Prediction Analysis of Microarray 50 (PAM50) Gene Expression Classifier Utilized in Indeterminate-Risk Breast Cancer Patients in Hungary: A Consecutive 5-Year Experience
Source: Genes (Basel). 2023 Aug 28;14(9):1708. doi: 10.3390/genes14091708 (PMC10530528; doi:10.3390/genes14091708)
Supplement: Supplementary file 1 [file genes-14-01708-s001.zip › genes-2497075-supplementary.pdf]

The Prediction Analysis of Microarray 50 (PAM50) gene expression classifier utilized in indeterminate-risk breast cancer patients in Hungary: a consecutive 5-year experience.

Magdolna Dank, Dorottya Mühl, Annamária Pölhös, Renata Csanda, Magdolna Herold, Attila Kristof Kovacs, Lilla Madaras, Janina Kulka, Timea Palhazy, Anna-Maria Tokes, Monika Toth, Mihaly Ujhelyi, Attila Marcell Szasz* and Zoltan Herold*

Table S1. Clinical and histopathological data of study participant who died during the observational period of the study.

Patient ID	Age at PAM50 sampling	Sample type	Survival time (month)	Primary or recurrent	TNM stage	IHC subtype	PAM50 subtype	ROR score (risk category)	10-year recurrence risk score
Patient 3	66.6	Surgical	39.6	Primary	T3N2M0	Luminal B	HER2-positive	81 (high)	43
Patient 10	84.6	Surgical	54.7	Primary	T1N1M1	Luminal B	Luminal B	39 (moderate)	15.33
Patient 27	60.9	Surgical	28.4	Recurrent	T1N0M1	HER2-positive	HER2-positive	67 (high)	19
Patient 39	62.9	Core biopsy	40.3	Primary	NA	HER2-positive	HER2-positive	66 (high)	28
Patient 40	33.1	Surgical	18.8	Primary	T1N2M0	Luminal B	Basal-like	9 (high)	43

IHC: immunohistochemistry; PAM50: Prosigna® Prediction Analysis of Microarray 50 gene expression testing kit; ROR: risk of recurrence; TNM: tumor – node – metastasis.

* These authors contributed equally to this study and shared the senior authorship.
